# Advanced Spectroscopic, Imaging, and Nanotechnology Tools for Diagnosing Fungal Diseases in Fruits

**DOI:** 10.1002/fsn3.71611

**Published:** 2026-03-08

**Authors:** Vanshika Adiani, Archana Mishra

**Affiliations:** ^1^ Food Technology Division Bhabha Atomic Research Centre Mumbai Maharashtra India; ^2^ Nuclear Agriculture and Biotechnology Division Bhabha Atomic Research Centre Mumbai Maharashtra India

**Keywords:** biosensors, fruits, fungal diseases, imaging techniques, nano‐diagnostic, spectroscopic techniques

## Abstract

Fruits are a critical component of the human diet, as they provide essential dietary nutrients that play an important role in the functioning of the human body and maintaining health. It is well‐known that consuming fruits has various benefits, including the prevention of chronic diseases, cancer, and cardiovascular disorders. Thus, wider availability and maintaining the quality of fruits are highly required. Around 25% of global crop losses reported annually are attributed to disease and pest infestations, as per the Food and Agriculture Organization. Fungal pathogens are a major cause of post‐harvest diseases, which significantly affect production and lead to economic losses. To address this, disease diagnosis at an early stage is crucial to enable timely monitoring, implementation of prevention techniques, and minimizing storage‐related losses. Various methods are available for early pathogen detection; spectroscopic and imaging techniques have been widely applied as they offer cost‐effectiveness, potential for real‐time analysis, and a non‐destructive nature of analysis. When integrated with advanced decision‐support tools, these instrumental techniques can enable rapid and accurate detection of fungal diseases in fruits. In recent years, nanotechnology has emerged as a promising approach, with a wide range of nanoparticles being utilized to develop nanobiosensors for various applications. This review also highlights recent advancements in the use of nanomaterials and nanoparticle‐based sensing systems for the detection of pathogens, providing an overview of their potential role in improving post‐harvest disease diagnostics.

## Graphical abstract

Schematic presentation shows various techniques applied for the detection of fungal diseases in fruits.

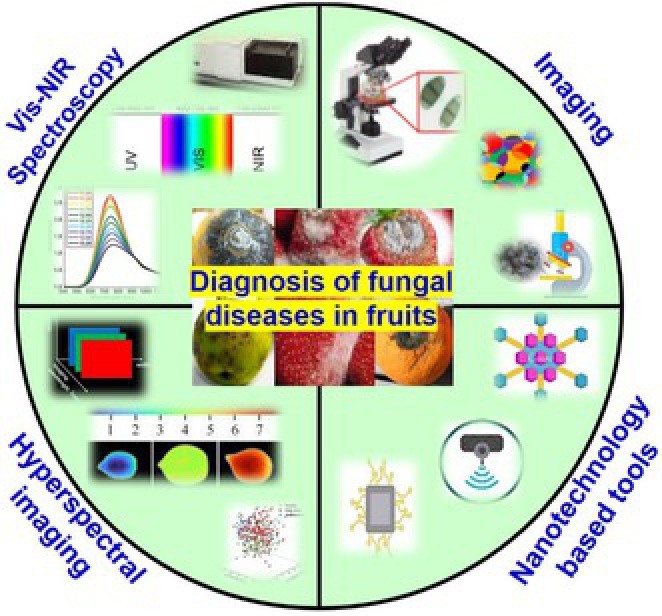

## 1 Introduction

**FIGURE 1:**
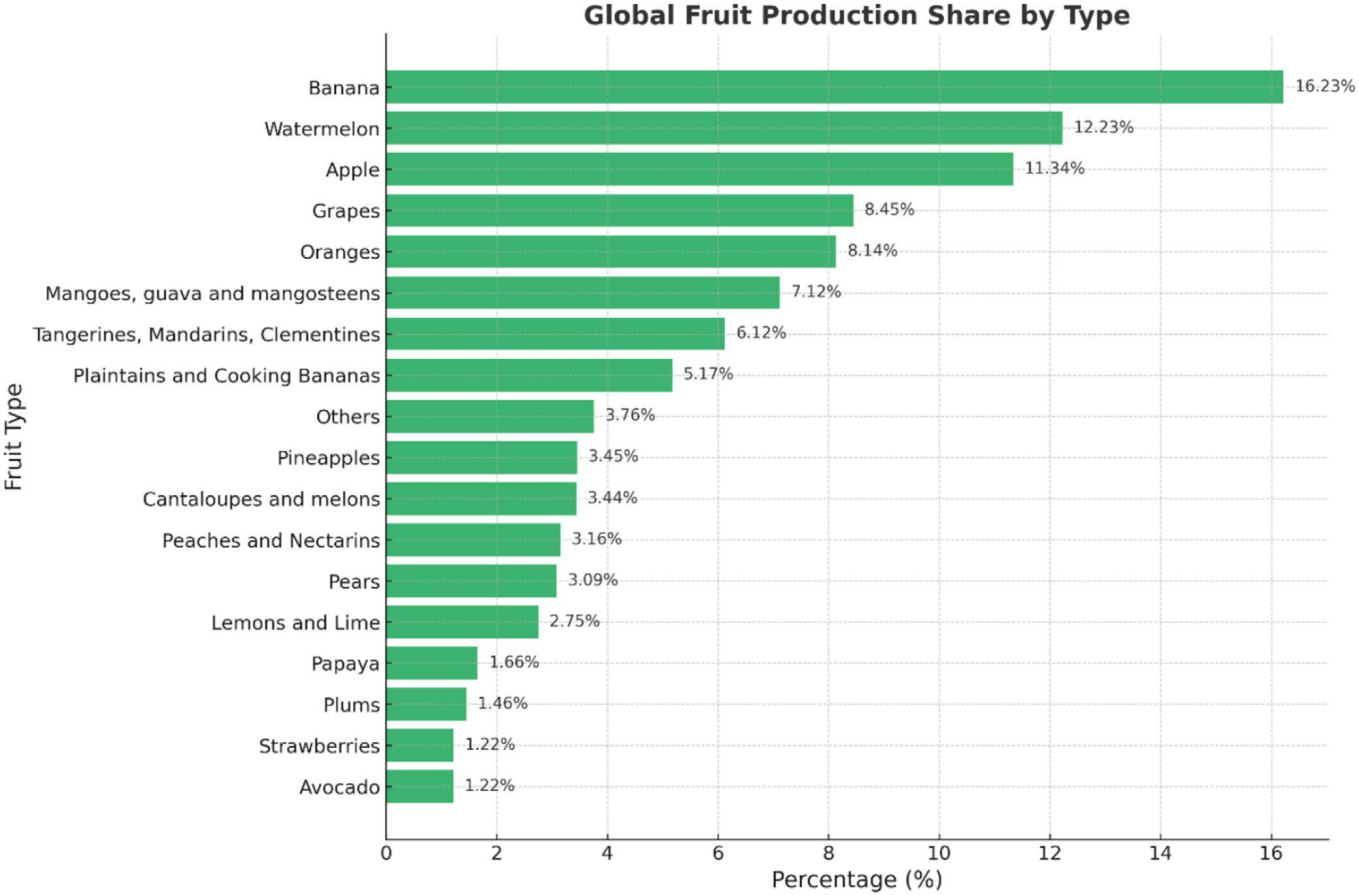
Production data of fruits worldwide (adapted from Statista 2023) (https://www.statista.com/statistics/264001/worldwide‐production‐of‐fruit‐by‐variety/).

Fruits are a major source of essential dietary nutrients in the human diet. Figure 1 represents the global production of different fruits worldwide. Fruit consumption has been found to be beneficial and effective in counteracting several chronic diseases such as cancer and cardiovascular diseases. Therefore, fresh fruit consumption is recommended for a balanced diet. Additionally, due to growing health awareness, there is a growing concern among consumers about the nutritional, sensory, and safety aspects of the food. More often, the quality of the product is tested by visual inspection and conventional methods that are generally destructive.

Post‐harvest disease incidence in fruits is often reported to be caused by fungal pathogens and leads to major production and economic losses. Therefore, it becomes imperative to detect developing disease in fruits in a timely manner and apply treatments suitable to prevent postharvest losses and also to prevent healthy ones getting infected. Thus, minimizing disease impact before full development (Pieczywek et al. 2018). Traditional methods are based on the direct observation of fungi. These include direct sampling of fungal fruiting bodies, incubation of substrata in moist chambers, laboratory culturing of endophytes, and particle plating. Classical sampling methods can be considerably more time consuming. Moreover, experts for taxonomic classification are required for classical methods (Sharma et al. 2016). Scouting involves physical monitoring of diseased or infected fruits which is currently widely utilized, however it is expensive, labor‐intensive and time taking process (Sankaran et al. 2010). Polymerase chain reaction (PCR) is one of the molecular techniques that when employed requires detailed sampling and processing to identify the plant diseases (Mirmajlessi et al. 2015). Following extensive research and development, precise, eco‐friendly, and non‐destructive techniques have been introduced to assess the quality of agricultural, food, and horticultural products such as fruits, vegetables, meat, fish, poultry, dairy items, eggs, and beverages (Vadivambal and Jayas 2011; Caballero et al. 2017; Suktanarak and Teerachaichayut 2017).

The assessment of quality and safety in agricultural produce, particularly fruits, has reached a cutting‐edge level with the introduction of innovative, non‐invasive sensing technologies that eliminate the need for human involvement. These advancements have not only improved production efficiency but also enabled precise measurement of individual quality attributes. In recent years, imaging techniques have been widely incorporated for food quality inspection. Spectroscopy combined with image processing has emerged as a powerful and cost‐effective approach for performing rapid, accurate, and reliable assessments. This plays a crucial role in quality and safety evaluations, effectively dealing with the sorting and grading of fruits based on various parameters (Rajkumar et al. 2012). Spectroscopic techniques along with advancements in computer technology and chemometrics have demonstrated promising capability for internal structure evaluation and chemical composition prediction in produce.

**FIGURE 2:**
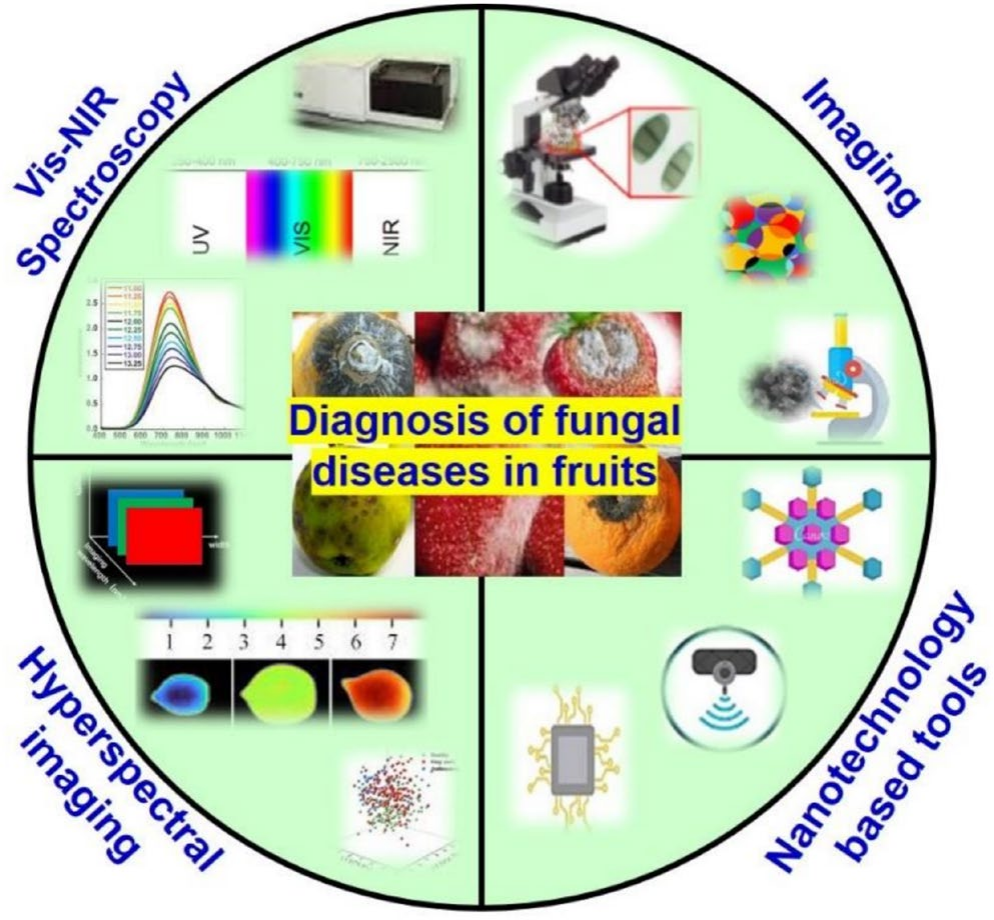
Schematic presentation shows various advanced techniques applied for the detection of fungal diseases in fruits.

Nanotechnology has emerged as an important field that has been widely applied to improve the sensitivity and specificity of diagnostic systems. The main objective of this review is to give an overview of the recent advances and applications of image processing and computer vision, vis/IR, fluorescence spectroscopy and hyperspectral imaging in combination with chemometrics for the detection and determination of fungal diseases in fruits (Figure 2). Also, nanobiosensors, nano sequencing techniques, nanomaterials, quantum dots, plant wearables and their role in early detection of plant pathogens are elaborated.

## 2 Fungal Diseases of Fruits

**FIGURE 3:**
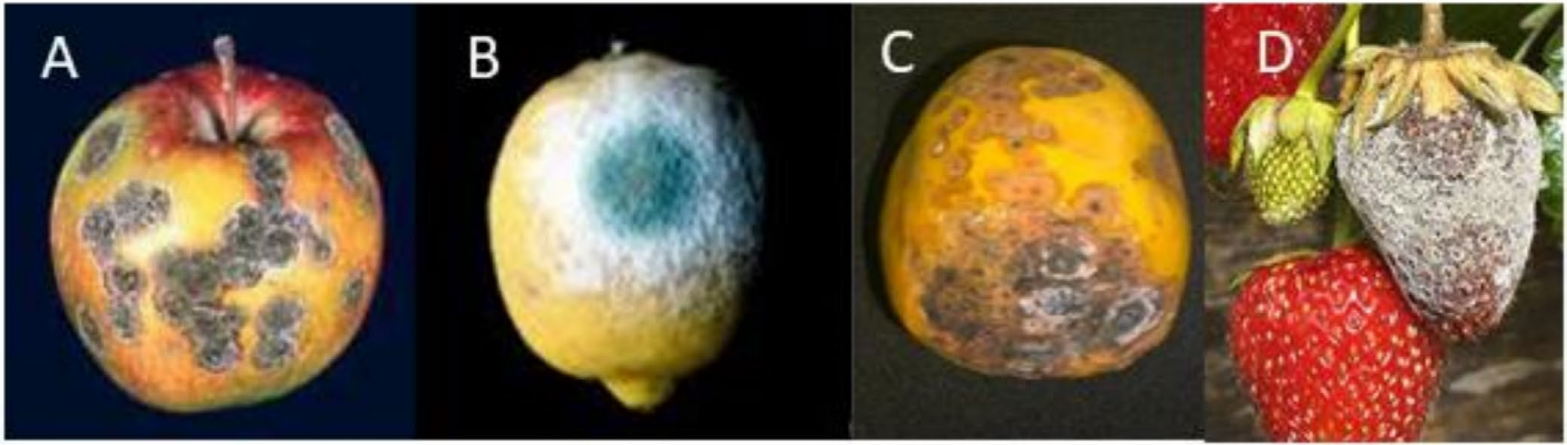
Schematic presentation of some representative fungal disease (A) Apple scab (B) Fungal lesion by *Penicillium digitum* infection on lemons (C) Anthracnose of papaya fruit (D) *Bortrytis* fruit rot on strawberry.

**FIGURE 4:**
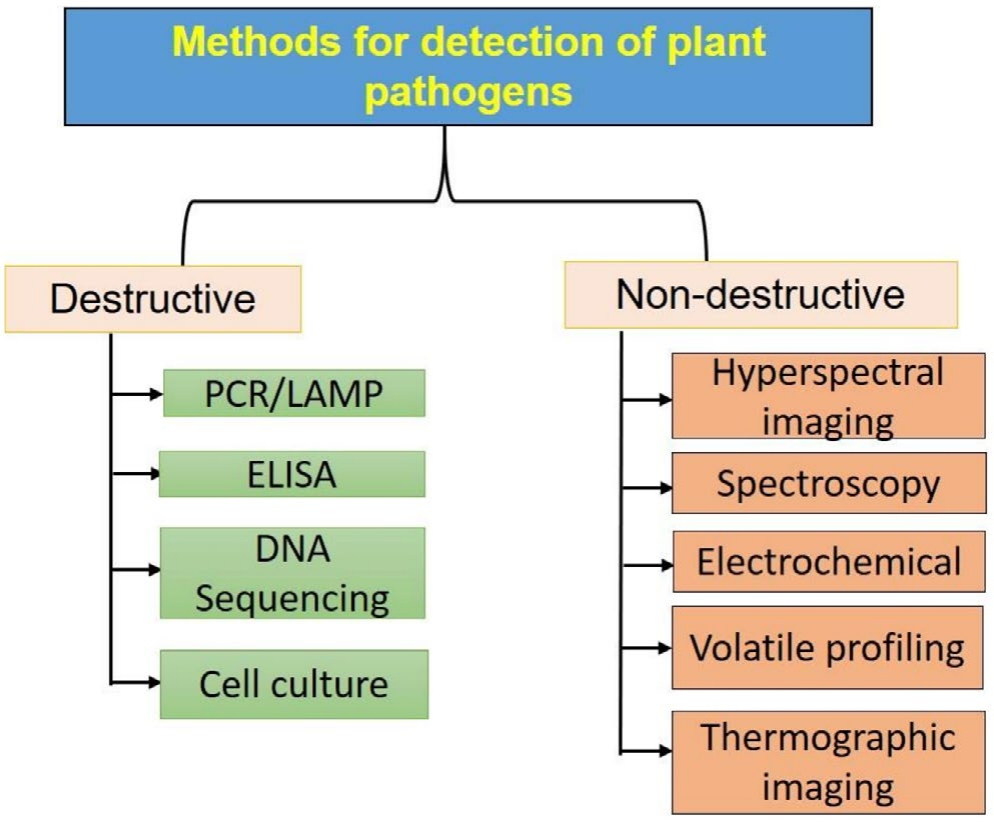
Various destructive and non‐destructive techniques for pathogen detection.

The fungal pathogens responsible for post‐harvest loss of crop are the inhabitants of the soil, air and water that belong to the microbial community and maintain the dynamic ecological balance. These can be introduced to the seed, during development or post‐harvest handling and distribution. Fungi produce extracellular pectinases and hemicellulases and are capable of colonizing and creating lesions (Miedes and Lorences 2004). Colonization and lesion development tend to occur more rapidly and frequently in plant tissues that are damaged or otherwise compromised. Physical injuries such as bruises, cracks, and punctures provide entry points for spoilage microorganisms to establish and proliferate. Lesions can form relatively quickly, often within a matter of days or weeks. The fungal pathogens are responsible for diseases such as anthracnose (characterized by formation of dark, depressed spots or lesions, frequently surrounded by a raised edge, on the affected fruit), rust (development of yellow spores), powdery mildew (white powdery growth), and soft rot (wet rot covered by a cottony white fungal growth) (Figure 3). The reported fungal pathogenic species in fruits mostly belong to the Basidiomycetes (genera: *Sclerotium*, *Rhizoctonia*) and Ascomycetes (genera: *Alternaria, Fusarium, Verticillium*). Due to severe economic losses associated with fungal diseases, in recent times, several destructive and non‐destructive methodologies are widely explored to detect fungal pathogens in horticulture produce, as depicted in Figure 4. This review also focuses on recent non‐destructive methodologies that can be integrated online and inline for rapid monitoring of the onset of fungal infections.

## 3 Chemometric Tools for Disease Detection

Several non‐destructive methodologies in recent times have been explored for disease detection. These methodologies when integrated with machine learning tools allows rapid real‐time analysis of disease onset. Several machine learning tools have been utilized which can be broadly classified as unsupervised or supervised techniques. Unsupervised techniques can differentiate different subset in a dataset without having any prior information about the different treatments given to a dataset (Valkenborg et al. 2023). While, supervised learning methods require prior information of the subsets in the dataset to generate prediction models that can later perform classification or regression (Adiani et al. 2014). Unsupervised technique widely utilized are clustering techniques (e.g., *k*‐mean clustering and hierarchical clustering) that group the data into clusters based on similarities and differences (Valkenborg et al. 2023). Alternatively, dimensionality reduction (e.g., Principal component analysis) is another widely used unsupervised technique that reduces features while preserving data information. Supervised techniques that are widely used for disease detection are support vector machines (SVM), partial least square regression discriminant analysis (PLS‐DA), Artificial neural network (ANN), and multiple layer perceptron (MLP). SVMs are widely utilized for classification modeling for the smaller dataset having nonlinear relationships and high‐ dimensional pattern recognition among variables (Ling and Heldman 2025). While, PLS‐DA is suitable for high‐dimensional data with a small data set to develop prediction models for classification. PLS takes into consideration the relationship between variables (Ling and Heldman 2025). Contrastingly, ANN takes into account non‐linear relationships and uses similar principles as a biological neural network. ANN is an interconnected mathematical system of unit called neurons that performs simulation to predict the outcome from the input variables. A most popular ANN model is the multilayer perceptron (MLP), which has a back propagation learning algorithm that builds the model based on the provided data (Adiani 2020). These models are widely applied by biologist to predict the disease onset in fruits when combined with suitable non‐destructive machine tools in a rapid way.

### 3.1 Image Processing and Computer Vision

Fungal contamination can occur at several stages such as pre‐harvest or post‐harvest during storage, and is largely influenced by environmental conditions like humidity and temperature. It is extremely vital in the early stage of developing infections to prevent spreading for better inspection control. Otherwise, disease may develop while transferring to the marketplace, thereby causing rejection by the buyer. One of the first most important properties that is measured as a quality index is the external color. Color index (CI) using HunterLab color space coordinates *L*, *a*, and *b* can be employed to measure the color. Imaging methods have been established as effective tools for evaluating the quality and safety of agricultural food items. Computer vision applications are slowly making their presence in the field of horticulture research, namely by classifying grains, detecting weeds, sorting fruits in fruit processing units, medicinal plant identification, and so forth. Image processing technology has found numerous applications in agricultural operations. These methods typically involve capturing digital images using a camera within a specific domain, followed by applying image processing techniques to extract relevant features for further analysis. Image processing applied on the image are segmentation, pre‐processing, feature extraction, and identification. Machine vision by image processing has been automated for the inspection of commodities such as apples, peaches, and tomatoes in respect to different physical parameters such as color, size, and shape in the industry (Hahn 2009).

Detecting defects in fruits through imaging remains challenging due to the natural variations in skin color across different fruit types, the wide range of defect types, and the presence of structures like stems and calyxes. To efficiently diminish such losses, it is essential to carefully analyze the observed characteristics and identify the relevant control factors. Table 1 summarizes the application of image processing for assessment of early detection of fungal disease in horticultural produce. Pujari and colleagues introduced a method for grading and classifying anthracnose fungal infection in mangoes (Pujari et al. 2013b). Various segmentation techniques were employed to isolate and quantify the affected fungal areas. Texture features were extracted using the Gray Level Run Length Matrix (GLRM), and an Artificial Neural Network (ANN) classifier was used to distinguish between fungal‐infected and healthy mango images. Leiva‐Valenzuela and Aguilera demonstrated a pattern recognition approach for the automated identification of stem and calyx ends, as well as the detection of damaged berries (Leiva‐Valenzuela and Aguilera 2013). Five feature selection algorithms were evaluated to identify the most effective features for subsequent classification and cross‐validation. Among the classifiers tested, support vector machine and linear discriminant analysis demonstrated the highest performance. Dubey et al. developed image‐based computer vision with model fruit as apple and carried out classification for various apple fungal diseases such as apple blotch, apple scab, and apple rot in image processing‐based approach (Dubey and Jalal 2012). *k*‐means clustering was used for segmenting the defect followed by feature extraction from these segmented images which was classified using multi‐class support vector machine into one of the classes. Dubey et al. implemented an adaptive method for detecting fruit diseases, which was validated through experimental testing (Dubey and Jalal 2014). An enhanced texture feature using the Improved Sum and Difference Histogram (ISADH) based on neighboring pixel intensity values was utilized. Their experimental results demonstrated significant accurate determination and automatic identification of fruit diseases. While image processing allows several advantages for disease detection based on visual defects or color changes, they cannot be efficiently utilized for classifying the diseased type. Gómez‐Sanchis et al. developed a hyper spectral computer vision system capable of detecting two types of *Penicillium* fungi at an early stage in citrus fruits (Gómez‐Sanchis et al. 2012). This system offers advantage that it uses spectral information beyond red, green and blue bands. This can be even extended to infrared spectrum, that subsequently allowed classification between different *Penicillium* infection earliest on onset of infection. Feature extraction was performed using Minimum Redundancy Maximal Relevance (MRMR) method. Multiple layer perceptron (MLP) was applied for classification which could successfully differentiate diseased fruits. In another study conducted on citrus fruit, a digital microscope was used to capture RGB images of grapefruits at different magnifications, encompassing six peel conditions under different diseased conditions such as greasy spot, canker, scab, melanose, and insect damage along with healthy peel. Regions of interest presenting either normal or diseased areas were extracted from the original RGB images and translated into the hue, saturation, and intensity color space. Afterwards, texture features were selected with the help of algorithms. The obtained results showed that above selected parameters along with microscopic imaging can effectively differentiate citrus peel conditions with 95% accuracy (Zhao et al. 2009). Habib et al. utilized machine vision to recognize and classify fruit disease in the papaya (Habib et al. 2020). As described, handheld mobile device was used to capture the images and the disease‐attacked regions were segmented by K‐means clustering algorithm. Afterwards, desired parameters were extracted and classified for disease and healthy fruit using support vector machine. A hybrid approach for detecting diseases in apple fruits was proposed by Samajpati and Degadwala (Samajpati and Degadwala 2016). During the experiments, a combination of completed local binary pattern (CLBP) and local ternary pattern with a Gabor classifier yielded better results. Specifically, the Gabor classifier using the CLBP descriptor achieved the accuracy rates of 100%, 80%, 80%, and 70% for detecting healthy apples (control), apple scab, apple blotch, apple rot, respectively. These studies demonstrate that image processing with effective segmentation methodologies and feature extraction along with machine learning classifier tools such as ANN, MLP, and SVM helps in early detection of disease condition in fruits.

**TABLE 1:** Overview of application of image processing in detection of fungal diseases in fruits.

Fruit sample	Disease	Feature extraction	Algorithm used	Classification accuracy	References
Mango	Anthracnose	GLRM	ANN		Pujari et al. 2013a
Apple	Scab, blotch and rot	*k*‐mean clustering	Multi class SVM	93%	Dubey and Jalal 2012
Apple	Scab, blotch and rot	*k*‐mean clustering	Multi class SVM	92.98% in RGB, 97% in HSV	Dubey and Jalal 2014
Berries		*k*‐means clustering	SVM		Leiva‐Valenzuela and Aguilera 2013
Citrus	*Penicillium* genus	MRMR	MLP	—	Gómez‐Sanchis et al. 2012
Citrus	Normal canker, scab, greasy spot, melanose	Reduced HIS (STEPDISC)	Discriminant analysis	95%	Zhao et al. 2009
Papaya	Black spot, powdery mildew, brown spot, phytophthora blight, and anthracnose	*k*‐mean clustering	SVM	90%	Habib et al. 2020

### 3.2 Visible Near Infrared (Vis–NIR) Spectroscopy

Visible and near‐infrared (Vis–NIR) spectroscopy techniques, with the ranges of 380–780 nm for visible and 780–2500 nm for near‐infrared light are recognized as a reliable method for assessing the physical and chemical characteristics of biological substances (Balage et al. 2015; Barbin et al. 2015). Optical spectroscopy is typically operated in various modes like diffuse transmittance, diffuse reflectance, and interactance modes, however reflectance mode is the most commonly used mode. Vis–NIR spectroscopy can be utilized for in food analysis can be utilized because of the interaction of electromagnetic radiation with the molecular components of food. Food items have several functional groups such as C‐H, N‐H, C‐O, and O‐H, which interact with light by absorbing energy and undergo vibrational transitions. This generates Vis–NIR spectral data that could be interpreted to assess the quality of food (Wei et al. 2014). Added advantage with Vis–NIR is the rapid measurement capability, low operational cost, and obtained spectral data that could be analyzed using both quantitative and qualitative methods. Also, this holds significant potential for online quality monitoring and multi‐constituent analysis, which enables the simultaneous assessment of various parameters. The advanced instruments currently available are capable of generating large volumes of data, which require efficient pre‐processing and robust evaluation to extract meaningful information. NIR spectroscopy is recognized as a rapid, non‐destructive, and efficient technique for assessing fruit quality which offers potential alternative to traditional methods (Kaddour and Cuq 2009; Alcalà et al. 2010).

Several applications of Vis–NIR have been developed for the classification and identification of fungal disease in fruit produce as summarized in Table 2. In 1998, Hirano et al. employed transmittance spectra in the range of 500 to 1500 nm to detect mold in kernel of peanut (Hirano et al. 1998). During the study, moldy peanuts were generated by inoculating a suspension of *Aspergillus flavus* spore. This study revealed that the ratio of transmittance of wavelengths at 700/1100 nm significantly differed between internally infected moldy and healthy kernels. Thus, this can be used for differentiating between healthy and moldy kernels even in the absence of visible external symptoms. Moreover, a strong linear correlation was identified between the 700/1100 nm transmittance ratio and the extent of triglyceride hydrolysis, indicating that changes in the spectra were due to the nutrient metabolism associated with fungal infection. Tallada et al. performed a study that categorized corn kernels based on the severity of fungal infection into four levels, ranging from healthy to severely affected by analyzing reflectance spectra within the 904 to 1685 nm range (Tallada et al. 2011). The Linear Discriminant Analysis (LDA) results demonstrated 85% of accuracy for distinguishing advanced‐stage infected samples from control, in comparison to 77% of average accuracy for early‐stage infections. This technique was also applied to detect decay in citrus fruits and reflectance spectra were collected across 650–1050 nm (visible—NIR) and 1000–1700 nm (NIR), spectral ranges. Low‐dimensional data representations were extracted and carried out as input feature vectors, afterwards it was analyzed using LDA. The obtained data showed an overall classification accuracy of 97.8% in decayed skin. This technique was also carried out in an online mode by Shenderey et al. to detect apples moldy cores using Vis–NIR mini‐spectrometer (400–1000 nm) that is installed in‐line. Herein, the system was equipped with four cells, each designed with rubber rings at the top and bottom to securely hold individual fruits (Shenderey et al. 2010). A fiber‐optic probe was positioned beneath each fruit cell. Analysis was conducted in transmittance mode, with each fruit scanned in approximately 1 s. The system demonstrated high classification accuracy, correctly identifying 92% of healthy apples and achieving 100% detection of deterioration when damage exceeded a level of 30. Additionally, the setup enabled high‐speed analysis, processing approximately five fruits per second. Studies demonstrated the potential of Vis–NIR spectroscopy for early detection of fungal disease in fruits. This technique holds promise with the ease of convenience it offers. It can be used in portable and handheld devices which can be deployed for the field application for rapid analysis of fungal infections in fruits. Despite of various advantages, a Vis–NIR technique also suffers with limitations such as sensitivity to light and penetration depth in fruit tissue thus limiting its use in subsurface infections especially when using reflectance mode. On the other hand, when this technique is utilized with complementary technique such as X‐ray and thermal imaging can also detect internal defects. Thus, Vis–NIR spectroscopy in combination with other techniques holds potential to be explored for real field applications to detect fungal pathogens.

**TABLE 2:** Overview of application of Vis–NIR in detection of fungal diseases in fruits.

Fruit sample	Disease	Absorption band	Algorithm used	Accuracy	References
Citrus	*Penicillium digitatum*	650 to 1050 nm; 1000 to 1700 nm	Image segmentation algorithm	97.8%	Lorente et al. 2015
Apple	Moldy core	400‐1000 nm	Canonical discriminant analysis of PLS‐R latent variables	92% healthy; 100% infected	Shenderey et al. (2010)
Sugarcane	Fiji leaf gall	11,000–4000 cm^−1^	PLS‐R	—	Purcell et al. (2009)
Peanut	Moldy peanut kernel	500 and 1500 nm	Ratios of 700/1100 nm	—	Hirano et al. 1998
Corn kernels	Fungal damage	904–1685 nm	LDA	85% infected	Tallada et al. 2011

### 3.3 Hyperspectral Imaging

Hyperspectral imaging combines conventional imaging with spectroscopic techniques to acquire both spatial and spectral information from fruits and vegetables, allowing for the assessment of essential quality attributes (Hahn 2009). While conventional spectroscopy such as Vis–NIR acquires the spectral information averaged over region, hyperspectral imaging gives both physical attributes by imaging and chemical information in spectral data at each pixel (Amoriello et al. 2025). During acquiring hyperspectral information, each pixel's reflectance is recorded over multiple wavelengths spanning the electromagnetic spectrum, often covering both the visible and infrared regions. While similar to multispectral imaging, hyperspectral imaging differs by scanning a wider wavelength range (with more spectral bands) for each pixel unit. This process generates a collection of pixel data indicating reflectance intensity across various wavelengths, resulting in a detailed image. Hyperspectral imaging is commonly applied to assess and monitor the quality of food items (Sankaran et al. 2010). This imaging technique can be operated in four modes namely reflectance, transmittance, fluorescence, and Raman scattering. The selection of mode largely depends on the application of interest. Diffuse reflectance imaging primarily examines the upper layers of biological materials, typically penetrating a few millimeters beneath the surface. It is frequently utilized to identify surface or near‐surface features in horticultural produce, including defects and signs of microbial spoilage (Mehl et al. 2004; Zhang, Li, Fan, et al. 2015; Zhang, Li, Huang, et al. 2015; Gómez‐Sanchís et al. 2008; Li et al. 2016). Transmittance imaging is beneficial for evaluating internal quality traits of samples, such as hidden defects, as it measures only the light that has passed entirely through the sample (Huang et al. 2013; Qin and Lu 2005). Fluorescence spectra of plant tissues typically exhibit emission peaks within the blue, green, red, and far‐red regions, covering wavelengths from approximately 400 to 800 nm (Buschmann and Lichtenthaler 1998). Blue and green emissions are generally associated with cinnamic acids, whereas chlorophylls are responsible for peaks in the red and far‐red areas (Buschmann et al. 2000). Monitoring fluorescence at these specific wavelengths is useful for assessing plant health (Lichtenthaler and Miehe 1997) and for inspecting the quality and safety of horticultural produce (Kim et al. 2002). Additionally, hyperspectral Raman imaging, an advanced 2D extension of traditional Raman spectroscopy, enables the detection of Raman scattering signals for detailed analysis (Matousek and Morris 2010). Raman imaging systems that are used for commercial purpose usually have small imaging areas, typically at the millimeter scale or smaller, making them suitable for microscopic applications. Hyperspectral Raman imaging has been utilized for generating chemical distribution maps of horticultural products (Qin et al. 2011). Integrated imaging modes such as reflectance and transmittance together have also been reported because they provide the advantages of conducting a simultaneous study of external (color, surface defects) and internal quality evaluation (soluble solid content, internal defects, texture) pioneered by Ariana and Lu (Ariana and Lu 2008a, 2008b). Hyperspectral image data are characterized by high dimensionality in both spectral and spatial domains. Typically, the raw hyperspectral data undergo five key processing steps: data pre‐processing, spectroscopic along with image analysis, modeling, and subsequent performance evaluation.

One of the major challenges in hyperspectral imaging for plant disease detection is selecting disease‐specific spectral bands and choosing an appropriate statistical classification algorithm, both of which depend heavily on the data acquisition setup used under field conditions. It has been employed to identify microbial spoilage or pathogenic contamination by distinguishing affected regions from healthy ones in food products, and for separating spoiled or infected items during sorting processes (Gómez‐Sanchís et al. 2008; Li et al. 2016; Siripatrawan et al. 2011). Table 3 presents a brief overview of the application of hyperspectral imaging for the early detection of fungal diseases in fruit samples. Gómez‐Sanchís et al. (2008) were the first to report the identification of early‐stage rot in citrus fruits (Gómez‐Sanchís et al. 2008). Decay caused by *Penicillium* sp. was identified through manual sorting under UV illumination. In order to avoid the use of UV light as it causes health risks to operators, a hyperspectral imaging system utilizing a liquid crystal tunable filter (LCTF) was developed, which attained a classification accuracy of 91% through the application of a regression tree algorithm. Hyperspectral reflectance imaging was employed by Gómez‐Sanchis et al. to identify and detect symptoms of rottenness in tangerines (Gómez‐Sanchis et al. 2012). Using the MRMR algorithm for optimal band selection, it was observed that the multilayer perceptron classifier outperformed the classification and regression tree method and achieved an accuracy of 98.30%. Further, Li et al. also utilized line‐scan hyperspectral reflectance imaging, spanning from wavelength 400 to 1000 nm, to differentiate between healthy tissues and canker‐infected areas, along with nine other peel diseases in ‘Navel’ oranges (Li et al. 2012). The combination of principal component analysis (PCA) and a two‐band ratio (I687/630) yielded a superior detection rate of 99.5% (training set), while 98.2% (testing set) when compared to the standalone two‐band ratio method, which achieved an accuracy of 84.5% and 82.9%, respectively. Using a similar spectral range, Qin et al. applied the spectral information divergence (SID) method to the average spectra of selected regions of interest (ROIs) to distinguish canker lesions from healthy tissue and five other peel disorders, achieving a 95.3% accuracy with no false negatives. Based on this, for real‐time grapefruit canker detection a compact hyperspectral reflectance imaging system was developed (Qin et al. 2012). However, this has its own limitations like it captured only a small portion of the fruit. Teena et al. demonstrated that hyperspectral imaging could effectively identify contaminated date fruits across the spectral range of 960 to 1700 nm, with 100% classification accuracy (Teena et al. 2014). The detection of early decay symptoms in citrus fruit caused by *Penicillium digitatum* was carried out using automatic detection. A spectral range from 325 to 1100 nm was utilized, and four key wavelengths, 575, 698, 810, and 969 nm, were identified. A pseudo‐color imaging combined with a thresholding technique was employed to develop an image segmentation algorithm for detecting decayed fruits, with 98.6% accuracy on the test (Li et al. 2016). Blasco et al., utilized multispectral computer vision, incorporating visible and non‐visible spectra, both of which include ultraviolet, infrared, and fluorescence for segregating citrus fruits (Blasco et al. 2007). Results demonstrated that anthracnose was effectively classified using NIR images, achieving an accuracy of 86%, while green mold was identified with 94% accuracy by fluorescence imaging. The stem‐end injury was 100% accurately classified with ultraviolet spectral data which demonstrated the effective use of different hyperspectral bands to detect/identify various aspects of a single issue. Also, Pearson and Wicklow evaluated the effectiveness of multispectral imaging in transmittance mode for differentiating corn kernels showing extensive discoloration because of fungal infection from asymptomatic kernels (Pearson and Wicklow 2006). The study utilized 11 interference filters centered at wavelengths of 780, 830, 870, 880, 890, 905, 920, 930, 960, 980, and 1020 nm, each with a 10‐nm bandwidth. Using three key features, the accuracy in classification modeling was achieved up to 93% for asymptomatic kernels while 90% for discolored ones. In another study, monitoring the pathogenic progression and early disease detection in pears of black spot by *Alternaria alternata* , remained challenging due to minimal visible changes during initial infection stages. To address this, models including *k*‐nearest neighbor (*k*‐NN), support vector machine (SVM), and partial least squares discriminant analysis (PLS‐DA) were developed and assessed for their effectiveness in early disease detection. The findings indicated that the SVM model can handle non‐linearity and can differentiate subtle class boundaries, thus achieving an overall accuracy of 97.5%, which was the most effective for the applied HSI method (Pan et al. 2019). Thus, these studies imply the utilization of hyperspectral imaging methodologies to effectively implement disease detection.

**TABLE 3:** Overview of application of hyperspectral imaging in detection of fungal diseases.

Fruit sample	Disease	Absorption band	Algorithm used	Accuracy	References
Citrus	*Penicillium digitatum*	325–1100 nm (575, 698, 810 and 969 nm, selected)	Image segmentation algorithm	98.6%	Li et al. 2016
Strawberry	*Botrytis cinerea*; *Collatotrichum acutatum*	450 nm and 2500 (19 wavelengths)	Backpropagation neural network (BNN) model	In inoculated and control fruit was higher than 97%	Siedliska et al. 2018
Strawberry	Fungal infections	400–1100 nm	SVM	92.59%	Liu et al. 2018
Mandarins	*Penicillium* sp.	400‐1000 nm	Classification and regression trees (CART) and LDA	91%	Gómez‐Sanchís et al. 2008
Tangerines	*Penicillium* sp.	400–1100 nm	MLP	98.3%	Gómez‐Sanchis et al. 2012
Oranges	Canterous tissue	400 to 1000 nm	Two band ratio I687/630	98.2%	Li et al. 2012
Citrus fruit	Canker spots	400 to 1000 nm	SID algorithm	95.3%	Qin et al. 2012
Date fruit	*Aspergillus flavus*	960–1700 nm	QDA and LDA	100%	Teena et al. 2014
Corn Kernels	Fungal infections	780–1020 nm	Neural network	93% asymptomatic; 90% infected	Pearson and Wicklow 2006
Blueberries	Fungal infection	685–1000 nm	PLSDA	98%	Huang et al. 2020
Grapevine bunches	*Erysiphe necator*	900–1700 nm	PLSDA	85.33%	Pérez‐Roncal et al. 2020
Pear	*Alternaria alternata*	400–1000 nm	SVM, K‐nearest neighbor, PLSDA	97.5%	Pan et al. 2019

### 3.4 Advancement and Challenges

While imaging and spectroscopic techniques offer several advantages in non‐destructive detection of fruit disease using chemometric tools. Current research also has to address the challenges such as robust methods that remain unaffected by ambient light conditions during disease detection (Pham et al. 2023). Further, data management for a large dataset with parallel processing for real‐time assessment is utmost necessary. Additionally, cost‐effective sensor technologies without performance deterioration require robust innovation. One of the important aspects that requires careful consideration is fruit variability associated with cultivar, growing condition, maturity, harvest time, and storage, which adds complexity. Therefore, feature selection with suitable methodologies adopted while including variations in the training set can increase efficiency. Certain advances for practical implementation may require fusion methodologies to improve discrimination and seamless integration needs to be attempted (Olorunfemi et al. 2024). Further, high‐speed imaging on sorting lines of a food processing industry that are integrated with instant data processing and decision‐making systems will be pivotal for its adoption.

## 4 Nanomaterials for Nanobiosensor

Nanotechnology is one of the popular areas of modern science, which has emerged as a rapidly advancing field with profound impact in various fields such as health care, food, agriculture, etc. Nanomaterials (size range of 1–100 nm) offer a number of advantages like high surface area, excellent chemical, optical, physical, and electrical properties which make them highly useful for analytical and sensing applications (Kerry et al. 2021). Due to their inherited characteristics (nano size and fast diffusion rates), nanoparticles interact with the target molecule in an efficient way which further helps to achieve faster response time and improved sensitivity. Moreover, their structural and surface properties can be precisely engineered to achieve desired morphologies and functional groups, allowing customization for specific applications (Mishra et al. 2025).

**FIGURE 5:**
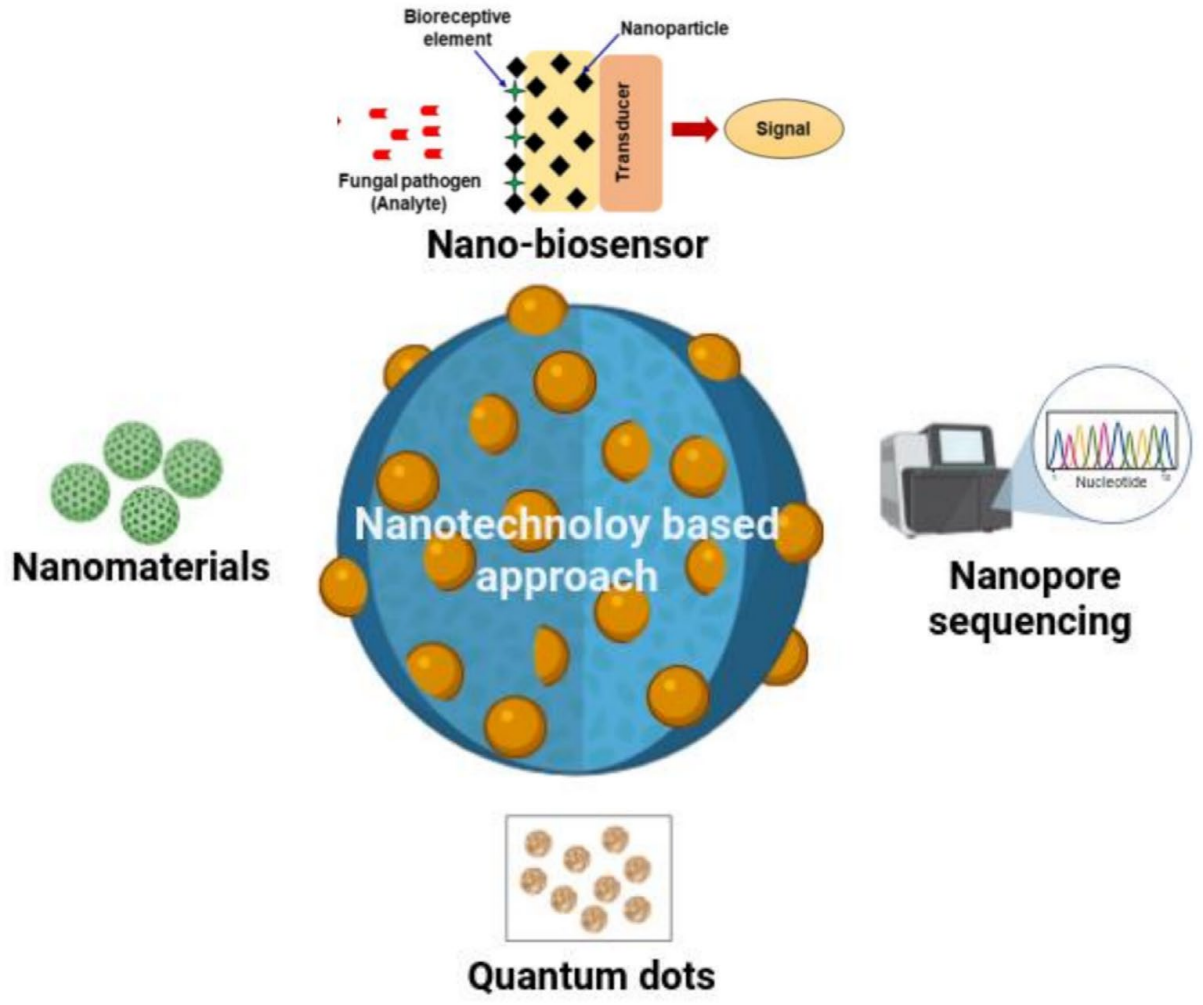
Various nanotechnology‐based approaches for detection of pathogens (the figure has been created using BioRender software).

In recent years, a wide range of nanomaterials have been employed for developing biosensors (Das et al. 2017; Mishra et al. 2017; Quesada‐Gonz'alez and Merkoçi 2018; Hu et al. 2018; Mishra, Kumar, et al. 2021; Ventura‐Aguilar et al. 2023). The association of nanomaterials with biosensing elements has significantly advanced agricultural diagnostics, providing field‐deployable, highly sensitive, and selective tools for pathogen detection. In this regard, nanobiosensors have shown great potential for the rapid and precise identification of phytopathogens responsible for crop losses. Current nanomaterials‐based pathogen detection systems utilize various approaches like nanobiosensors, nanopore sequencing, functional nanomaterials, quantum dots (QDs), and so forth (Figure 5). These emerging technologies comprise a new generation of analytical tools that has potential to revolutionize the detection of pathogens.

### 4.1 Nanobiosensors and Its Application

**FIGURE 6:**
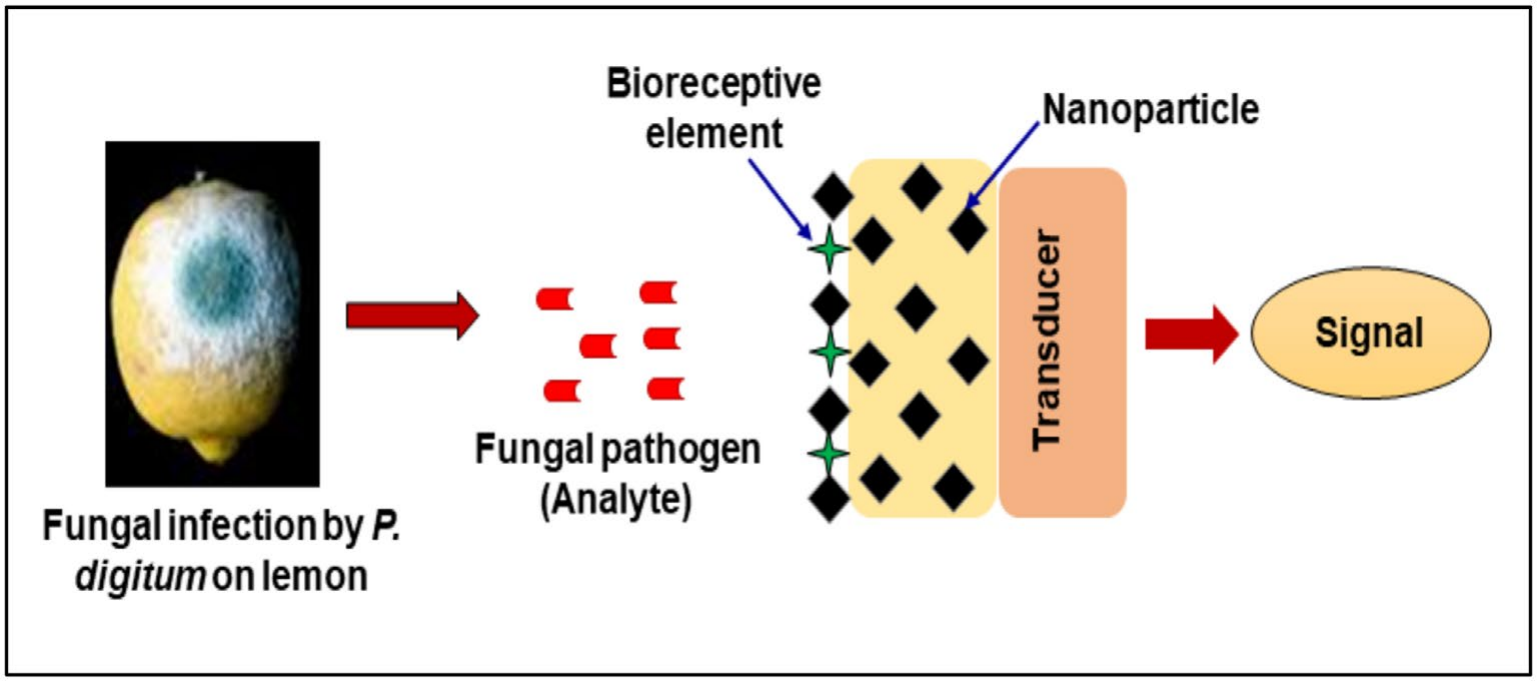
Schematic presentation showing a fungal disease on lemon fruit and its detection using a nanobiosensor‐based approach.

Advanced imaging techniques like hyperspectral and thermographic imaging have been widely utilized for the early detection of plant pathogens and the monitoring of plant health. Although these methods offer non‐destructive and large‐scale assessment, they have their own limitations, like susceptibility to variations in environmental conditions and a lack of molecular specificity (Giri et al. 2023; Li et al. 2014; Rousseau et al. 2015). To address these issues, nanotechnology‐based biosensing platforms have emerged as a powerful tool. In recent years, nanobiosensors and biosensors have been reported for pathogen detection (Chaturvedi et al. 2025; Khater et al. 2017). Nanobiosensors and biosensors are analytical devices that integrate a biological recognition element with a physicochemical transducer (Figure 6). The biorecognition component is mainly an enzyme, antibody, aptamer, or oligonucleotide that selectively interacts with the target analyte, while the transducer converts this molecular recognition event into a measurable electrical, optical, or electrochemical signal (Chaturvedi et al. 2025; Ventura‐Aguilar et al. 2023). The specificity of the biosensor depends on the biorecognition element, while its sensitivity can be significantly enhanced by the incorporation of nanomaterials that act as transducers or signal amplifiers. Also, nanomaterials can be applied as a support for immobilization of biorecognition elements; nanomaterials could provide a suitable microenvironment and improve the stability of biorecognition elements (Mishra, Kumar, et al. 2021). Various classes of nanomaterials like inorganic nanoparticles, carbon‐based nanostructures, and quantum dots have been successfully utilized to improve biosensor performance. Among inorganic nanomaterials, silver, gold, silica, and metal oxide nanoparticles have been widely explored for immobilizing biomolecules and amplifying detection signals (Wu et al. 2013; Darr et al. 2017; Mishra, Kumar, et al. 2021). Self‐assembled nanostructures have also been employed in biosensing, where nanoparticles conjugated with oligonucleotides (Dubertret et al. 2001; Cho and Ku 2017; Lau et al. 2017) or proteins (Li et al. 2020) facilitate selective and rapid detection of target biomolecules. For instance, a platinum–IgG antibody‐based nanosensor demonstrated improved sensitivity for detecting plant‐associated bacteria in soil and carrot samples (Ahmad et al. 2012).

Among others, silica nanoparticles have gained particular attention owing to their ease of surface modification, chemical stability, and high loading capacity for biomolecules (Mishra 2019; Mishra et al. 2025). These materials have been effectively utilized for immobilizing enzymes and other biocomponents (Mishra et al. 2014, 2020, 2023; Shukla et al. 2020; Lahiri et al. 2021; Mishra, Pandey, et al. 2021). In one of our previous studies, a microplate‐based biosensor incorporating silica nanoparticles was developed for the detection of the pesticide methyl parathion. The inclusion of silica nanoparticles significantly enhanced both the sensitivity and storage stability of the biosensor (Mishra et al. 2017). Similarly, silica nanoparticles functionalized with a Ru (II) complex and secondary antibodies were shown to enable highly sensitive detection of plant pathogens (Yao et al. 2009).

Gold nanoparticle‐based systems have also been extensively explored in this context. For example, a gold nanoparticle–single‐stranded DNA (ssDNA) composite was developed for the detection of *Ralstonia solanacearum* genomic DNA, demonstrating excellent specificity and sensitivity (Khaledian et al. 2017). Similarily, a screen‐printed electrode‐based sensor associated with silica microsphere and gold nanoparticles were applied for the detection of *E. coli* DNA and a linear range from 1 × 10^−16^ to 1 × 10^−11^ was achieved (Ariffin et al. 2020). Overall, nanoparticle‐based sensing systems (nanobiosensors) hold immense potential for the rapid and precise recognition of fungal and microbial pathogens. Table 4 summarizes representative studies employing various nanoparticles for the detection of fungal, bacterial, and viral pathogens.

**TABLE 4:** List of pathogens detected using nanoparticles‐based approaches.

Analyte (pathogen)/fruit	Mechanism of detection	Nanoparticles/biocomponent	LOD/accuracy	References
*Aspergillus* and *Rhizopus* fungi/Strawberry	E‐nose	N and B‐dopped Muti walled carbon nano tubes (MWCNTs)/−	—	Greenshields et al. 2016
*Phytophthora infestans/*Tomato	Chemiresistive sensor array	rGO and AuNPs/−	> 97% accuracy	Li et al. 2021
*Phytophthora cactorum*	Screen printed carbon electrode (SPCE)	TiO_2_ and SnO_2_ nanoparticles/−	LOD: 35–62 nmol L^−1^	Fang et al. 2014
*P. infestans/*Tomato	Optical detection	AuNPs/(Cys)‐capped	0.4 ppm	Li et al. 2019
*Phytophthora infestan/*Potato	Lateral Flow Assay	AuNPs/Streptavidin‐biotinylated T and C	0.1 pg. μL^−1^	Zhan et al. 2018
*X. arboricola pv. Pruni*/stone fruits and almond	Lateral Flow Assay	Carbon nanoparticles Polyclonal antibodies 2626.1‐WC	10^4^ CFU mL^−1^	Lopez‐Soriano et al. 2017
*Phakopsora pachyrhizi/*Soyabean	Fluorescence	Fluorescent nanoparticles IgG antibodies	2.2 ng mL^−1^	Miranda et al. 2013
*Xanthomonas campestris/*Brassica	Colorimetric	Gold nanoparticles (AuNPs)	10^2^ CFU mL^−1^	Peng and Chen 2018
*Yellow leaf* curl *virus/*Tomato	Surface plasmon resonance	AuNPs/reverse primer (20‐mer)	5 ng μL^−1^	Razmi et al. 2019
*Leafroll virus/*Potato	Lateral Flow Assay	AuNPs and Silver/Anti‐PLRV antibodies	0.2 ng mL^−1^	Panferov et al. 2018
*Penicillium digitatum*/Orange	Whole‐cell based biosensor	Genetically modified *E. coli* for detection of VOCs	—	Chalupowicz et al. 2020
*Aspergillus* and *Penicillium*	Colorimetric biosensor	Fe_3_O_4_/graphene oxide‐AFB1 aptamer for aflatoxins B1 (AFB1)	Aflatoxins B1 (detection limit 5–250 ng mL^−1^) and ochratoxin A (detection limit 0.5–80 ng mL^−1^)	Zhu et al. 2020

### 4.2 Quantum Dots (QDs)

Quantum dots (QDs) are extensively explored nanomaterials for a wide number of applications (Yadav et al. 2021). Due to their excellent photophysical properties, they are opted for the development of optical nanosensors. Previously, QDs have been applied as biosensors for plant imaging and disease detection (Wang et al. 2016). CdSe–ZnS core–shell QD with 3‐mercaptopropionic acid coating was prepared, and it was observed that it was rapidly taken up by fungal hyphae (Rispail et al. 2014). In comparison to conventional semiconductor QDs, the carbon dots (CDs) prepared from plant‐based products and proteins like bovine serum albumin have low cytotoxicity and high biocompatibility and thus are preferred for in vivo imaging (Yadav et al. 2021). Also, fluorescent CDs could be applied for the detection of phytopathogens through cell tracking and labeling of DNA (Shoala 2019). Further, a fluorescence resonance energy transfer (FRET) based complex system comprising CdTe QDs conjugated with coat protein (CP) along with CP‐labeled rhodamine dye was investigated for *Citrus tristeza* virus detection (Safarnejad et al. 2017). Thus, functionalized QDs could be useful for plant disease management and sensing of plant pathogens.

### 4.3 Nanopore Sequencing

High‐throughput sequencing technologies have advanced the accurate and sensitive detection of plant pathogens. Among these, nanopore sequencing represents a promising platform as it enables real‐time analysis of nucleic acids. In this technique, a motor protein was used to transport RNA or single‐stranded DNA through a nanopore and generates electronic current signals which led to the detection of pathogens (Eisenstein 2013). In comparison to previous sequencing techniques, this offers many advantages like fast running times, scalable genome mapping, real‐time and long reads of sequence data and small sample loadings (Watson et al. 2020; Wilson et al. 2022; Charalampous et al. 2019; Chalupowicz et al. 2019). Various researchers have employed nanopore sequencing technique to study the diagnosis of plant pathogen. A protocol was developed using a handheld sequencing system for the detection of various plant biotic stresses like fungi, viruses, bacteria, and phytoplasma including *P. digitatum* fungus in lemon (citrus fruits) and *S. lycopersicum* fungus in tomato (Chalupowicz et al. 2019). Also, using MinION, genome mapping of several plant viruses was successfully performed by Filloux et al., demonstrating the capability of this system for rapid diagnostic (Filloux et al. 2018).

Although it offers various advantages, the applicability of nanopore sequencing systems is limited due to poor discriminatory ability between highly similar sequences and high per‐read error rate (Filloux et al. 2018). Thus, constant efforts are required to be made to develop more powerful and reliable sequencing systems for the detection of plant pathogens.

### 4.4 Microneedle Patches

Conventional diagnostic techniques like enzyme‐linked immunosorbent assay (Sakamoto et al. 2018) and polymerase chain reaction (Bergeron et al. 2019) are widely used for the identification of plant pathogens. Although these methods offer several advantages, their application is often limited by long assay times, complex sample preparation procedures, and the requirement for expensive instrumentation (Lau and Botella 2017). These limitations can potentially be addressed by leveraging the unique physicochemical and functional properties of nanomaterials. In this regard, micro‐analytical systems like microneedle (MN) patches could be a better alternative.

In the field of health care, MN patches are well suited for various applications like non‐invasive delivery of therapeutic molecules along with in situ monitoring of clinical parameters. While, MN patches are widely used in the health care and nanomedicine however; limited research is available on application of microneedle for sensing of plant pathogens. Recently, a polyvinyl alcohol polymer‐based microneedle patch was prepared for real‐time, sensitive, and rapid recognition of *P. infestans* fungus, which is responsible for late blight disease in potato and tomato (Paul et al. 2019; Li et al. 2020). However, this system has poor specificity in isolation of nucleic acid. Thus, there is a need to work on developing microneedles using engineered nanomaterials which will help in highly specific, rapid, and efficient sensing of pathogens, leveraging the way for next‐generation diagnostic tool to manage post‐harvest loss.

### 4.5 Plant Wearable

The advancement of micro‐electro‐mechanical systems (MEMS) has enabled the development of implantable and wearable electronic devices capable of on‐demand monitoring of plant pathogens. This also applies for detection of various associated parameters associated with pathogen or plant's produce like volatile organic compounds (VOCs) and biomarkers. In recent years, plant wearables have emerged as an innovative diagnostic tool which can be attached directly to the plant's part and used for continuous monitoring of physiological and environmental changes (Li et al. 2020). For instance, a graphene‐based wearable sensor was developed which can monitor the evaporation of water from leaves and changes occurring in the electrical resistance of graphene under various humidity conditions were recorded which shows the plant behavior under abiotic stress (Oren et al. 2017). Similarly, another plant wearable was developed by Nassar et al. (2018) for studying the local microclimate of the plant and its growth. Furthermore, a biocompatible complementary metal‐oxide‐semiconductor (CMOS) flexible circuit was prepared by Lei et al. that has the ability to be attached on the rough surface of plants, demonstrating excellent mechanical flexibility and adhesion (Lei et al. 2017).

While wearable systems are already well established in human healthcare applications, their use in plant health monitoring is gaining increasing attention for monitoring abiotic and biotic stresses. However, despite these advances, the application of plant wearables for the detection and monitoring of fungal pathogens in fruits and other crops remains underexplored. Therefore, further research is required to develop next‐generation plant wearables integrated with nanosensors and biocompatible materials to enable real‐time, non‐invasive, and precise detection of plant pathogens.

## 5 Conclusion

Post‐harvest fungal/microbial contamination represents a major cause of global food loss, accounting for nearly one‐third of total agricultural production losses. Ensuring food safety is therefore essential for maintaining economic stability and supporting international trade. Conventional diagnostic methods, although effective, are limited by their complexity, high cost, and time‐consuming nature. Recent advances in non‐destructive detection techniques such as image processing, computer vision, near‐infrared spectroscopy, hyperspectral imaging, and nanobiosensor offer promising alternatives for rapid, real‐time, and non‐invasive monitoring of fungal infections in fruits. Each technique, however, presents inherent challenges, including the need for complex chemometric modeling, data handling, and high equipment costs. Despite these limitations, continuous refinement of these imaging and analytical systems will enable faster, more accurate, and cost‐effective identification of fungal spoilage in horticultural produce.

## 6 Future Prospective

Cheap sensing technologies and fusion technologies along with chemometric tools will allow rapid assessment of fungal disease in fruits. Effective strategies for feature extraction considering the inherent variation in fruit and mitigation strategies for light variation under ambient conditions are a must for image processing. Large data processing and wireless transmission of data and real‐time chemometric evaluation along with AI will ensure future deliverance of disease detection in a non‐destructive way. In the context of nanotechnology, an efficient biosensing platform will improve pathogen detection and portability. With the advances and continued research in areas of field deployable nanobiosensors, microneedle‐based DNA extraction systems, and portable nanopore sequencing will enable on‐site diagnostics with sensitivity and specificity. Real‐time monitoring of pathogen incidence can also be realized by smartphone‐assisted and wearable plant sensors. The convergence of nanobiosensing, non‐destructive imaging, and computational analytics is expected to drive the next generation of smart diagnostics, enabling timely detection, prevention, and management of fungal diseases, thereby ensuring food safety and sustainability in global agriculture.

## Author Contributions

**Vanshika Adiani:** conceptualization, writing original draft, review and editing of the manuscript. **Archana Mishra:** conceptualization, writing original draft, review and editing of the manuscript.

## Funding

The authors have nothing to report.

## Conflicts of Interest

The authors declare no conflicts of interest.

## Data Availability

No datasets were generated during this study.
